# Experimental determination of the dielectric constant of wheat grain and cluster straw in different moisture contents

**DOI:** 10.1002/fsn3.1350

**Published:** 2019-12-23

**Authors:** Mohammad Jafari, Gholamreza Chegini, Behrooz Rezaeealam, Amir Abbas Shaygani Akmal

**Affiliations:** ^1^ Department of Agrotechnology College of Abouraihan University of Tehran Tehran Iran; ^2^ Lorestan Agricultural and Natural Resources Research and Education Center, AREEO Khorramabad Iran; ^3^ Department of Electrical Engineering Lorestan University Khorramabad Iran; ^4^ School of Electrical and Computer Engineering College of Engineering University of Tehran Tehran Iran

**Keywords:** cluster straw, dielectric constant, electrical capacity, wheat grain

## Abstract

One of the new methods in the removal of impurities from the wheat grains is the electrostatic separation of particles based on the differences in their conductivity and permittivity. The moisture content of particles directly influences their electrical properties. In this study, by developing a cylindrical capacitor in an electrical circuit, the electrical capacitance of wheat grain and cluster straw particles in two varieties at four levels of moisture content (8%, 14%, 20%, and 25%) and five frequency levels (1, 10, 20, 100, and 500 kHz) is estimated and their dielectric constant is calculated. Results showed that increasing the moisture content of straw particles and reducing the frequency compared to the seeds had significant effects on dielectric constant. The dielectric constant of grain particles was higher than the straw at lower moisture content and in high values of moisture content, the dielectric constant of straw particles was higher than the seeds.

## Graphical abstract

This study shows that increasing the moisture content of straw particles and reducing the frequency compared to the seeds had significant effects on dielectric constant. The dielectric constant of grain particles was higher than the straw at lower moisture contents and in high values of moisture content, the dielectric constant of straw particles was higher than the seeds.

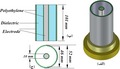

## 1 INTRODUCTION

Wheat is one of the main sources of food that is cultivated in most parts of the world. According to the FAO reports, the annual production of wheat in all over the world and Iran is 750 and 11.1 million tons in 2016, respectively (FAO, 2016).

Knowledge of the physical properties of agricultural products is useful in the design of agricultural machines. Determination of the physical properties of agricultural products results in the development of many instruments. These properties are important in the design and construction of machines used for planting and harvesting of agriculture products such as grains (wheat and barley), as well as transportation and processing (Baryeh, 2002). One of the new methods for separating seed from impurities is the use of electrostatic separators. In these separators, the differences in the electrical properties of the seeds are used to separate seeds that are not separable by conventional methods. These separators can separate seeds that have the same physical properties as the original seed, in addition to improve their germination percentage. The factors that affect the performance of this separation are the dielectric constant and electrical resistance of the particles. The dielectric or permeation coefficient is the property that a material exhibits in an electric field (Nelson & Trabelsi, 2017).

Information on the dielectric constant of grain has resulted in the development of a capacitance type grain moisture meter. Knowledge of the dielectric properties of seeds, grains, and other products is useful in many applications, including the prediction of the behavior of materials when exposed to electrical fields. The dielectric properties of food and agricultural products are basically used to describe the thermal behavior of materials when exposed to high‐frequency electrical fields in dielectric thermal processes and the development of appropriate techniques for the rapid moisture content determination of agricultural products (Nelson & Bartley, 2000).

The moisture content of agricultural materials is an important indicator of the changes in their dielectric properties which is sensed through correlations with the electrical characteristics or dielectric properties, so obtaining these values in a certain moisture content determines the relationships between them (Nelson, 2010; Soltani, Alimardani, & Omid, 2010).

Several methods have been proposed to predict the moisture content of agricultural products. Among these methods, electrical measurement is a low cost, rapid, and effective technique and instruments based on this technique are subject to less errors that arise from nonuniform distribution of moisture and physical contact with the materials under test. The dielectric constant of many agricultural materials has been investigated. Furthermore, many researchers have described the correlation between moisture content and dielectric constant of grains and seeds.

A common method for determining moisture content is to dry the material in the oven, which may be a destructive and time‐consuming method to remove all moisture. Although waveform spectrometry is a more appropriate method for determining the moisture content of agricultural materials, this method requires expensive and complex protocols (Edwards, Pirgozliev, Hare, & Jenkinson, 2001; Trabelsi, Kraszewski, & Nelson, 1998). Burton and Pitt (1929) developed a dielectric method for determining moisture content of four wheat cultivars. Casada and Armstrong (2008) measured the moisture content of wheat with a fringing field capacitive sensor. They extracted the linear calibration equations over a temperature range of 10°C to 30°C (Soltani et al., 2010). Prasad, Das, Ahmad, and Singh (2010) used dielectric constant of soybean at 2.45 GHz and 24°C to predict its moisture content. They introduced a quadratic equation between moisture content and dielectric constant of soybean. Sacilik and Colak (2010) determined the dielectric properties of corn seeds as functions of moisture content, bulk density, and frequency. They reported the moisture content was the most significant factor affecting the dielectric properties of corn seeds. Also, dielectric constant and loss factor increased with increasing moisture content.

The complex frequency‐dependent absolute permittivity of the material ε* is obtained with ε* = *ε_r_ε*_0_ = *ε*′ − *jε*″ where *ε*′ is the dielectric constant and *ε*″ is the dielectric loss factor that are called the real and imaginary parts of relative permittivity, respectively, and *ε*_0_ is the vacuum permittivity equal to 8.854 × 10^−12^ F/m. The relative permittivity of each material (*ε_r_*) is defined as the ratio of absolute permeability to the vacuum permittivity (Nelson & Trabelsi, 2017; Soltani & Alimardani, 2011a). Measurements of permittivity coefficients and dielectric properties of many kinds of grain and seed in the frequency range from 1 to 50 MHz have revealed high correlations between the grain and seed moisture content and their dielectric properties.

The most used instruments to determine dielectric properties of agriculture materials include the parallel plate capacitor, coaxial probe, waveguide, resonant structure, inductance, capacitance—resistance meter (LCR meter), impedance analyzer, and scalar and vector network analyzer (Ragni, Gradari, Berardinelli, Giunchi, & Guarnieri, 2006). Many commercial instruments have been developed for measuring grain and seed moisture content. Up to 80 years ago, the grain moisture content meter was often used at 1–20 MHz frequencies by parallel plate capacitive or coaxial sample holder where grain samples were placed between the electrodes (Lawrence & Nelson, 1998; Nelson, 1965). Trabelsi, Nelson, and Lewis (2009) measured the dielectric properties of shelled peanuts to estimate its moisture content. They carried out the experiments at temperatures ranging from 1 to 38°C and frequencies ranging from 8 to 14 GHz. They obtained the best results at 10 GHz frequency and proposed that equation was able to predict the moisture content as a function of temperature and dielectric properties without knowing the bulk density.

In this study, by developing a cylindrical capacitor in an electrical circuit, the electrical capacitance of wheat grain and cluster straw particles in two varieties (Chamran2 and Mehregan) in four levels of moisture content (8%, 14%, 20% and 25%) and five frequency levels (1, 10, 20, 100, 500 kHz) is measured and their dielectric constant is calculated.

## 2 MATERIAL AND METHODS

The samples of wheat in the form of spikelet from two cultivars of Chamran2 and Mehregan were prepared from the farm of Lorestan Agricultural Research Center and were threshed and cleaned manually. Straw masses mostly included lemma and palea and less amount of straw, rachis, and pedicel. Initial moisture content of samples which included grain and straw were measured by oven method. The moisture content of the samples was increased by flooding method in distilled water in variable time (5–25 min) to obtain 5 levels of moisture (Khoshtaghaza & Mehdizadeh, 2006). The excess water was removed from the wetted samples at each interval. For moisture content measurement, a part of each sample was weighted and transferred to oven at 104°C for 48 hr. The remaining part was kept in refrigerator at 4°C for 72 hr for equalization.

### 2.1 Circuit and measurement instrument design

**Figure 1:**
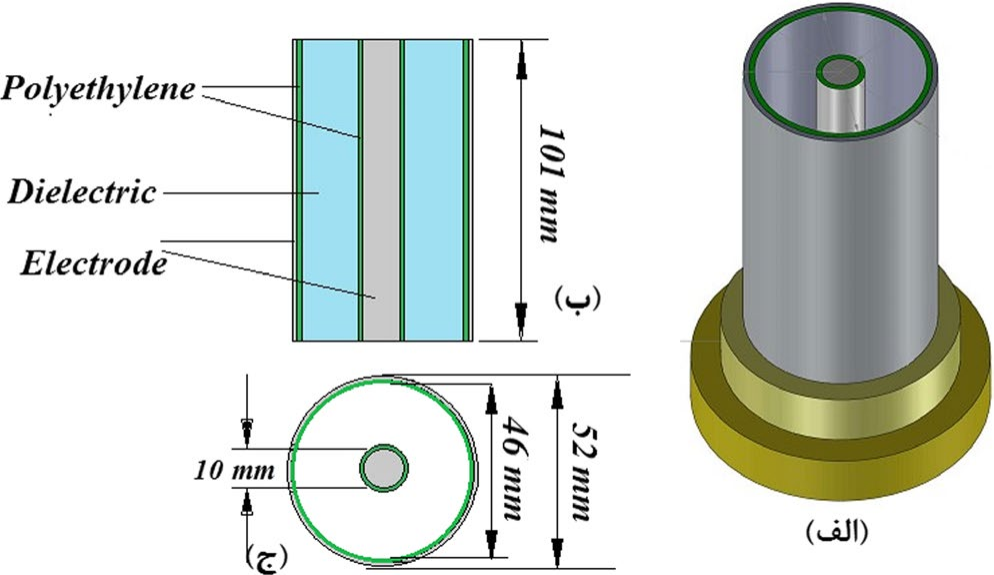
Schematic diagram of cylindrical capacitive sensor used for seed moisture content determination

To measure the dielectric constant of grain and straw at 5 levels of frequency at various moisture content, an instrument based on capacitive technique that include coaxial sample holder was designed and developed based on Soltani and Alimardani (2011b). The external electrode was an aluminum cylinder with 52 mm diameter, 3 mm thickness, and 100 mm height, and the internal electrode was an aluminum rod in the center with 10 mm diameter (Figure 1).

**Figure 2:**
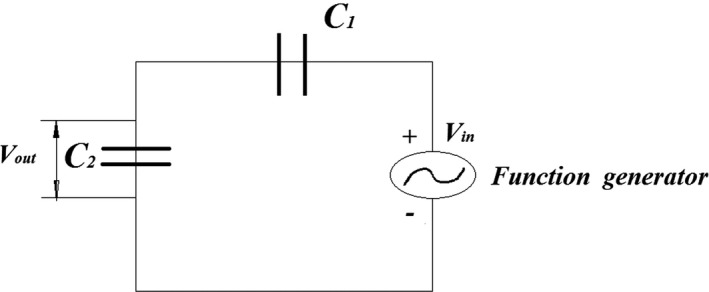
Voltage divider circuit used for capacitance measurement

Inner surface of the cylinder electrode and the rod electrode was coated by polyethylene insulate. The assemble capacitor was installed to the circular base. To measure the capacitance of sensor, a voltage divider circuit was used (Figure 2). This capacitor circuit, in addition to a cylindrical capacitor had a function generator and a parallel specific capacitance that was placed to the circuit in form of series and an oscilloscope as the display of the values of voltage variations at different frequencies.

### 2.2 Experiments

#### 2.2.1 Dielectric calculation

**Figure 3:**
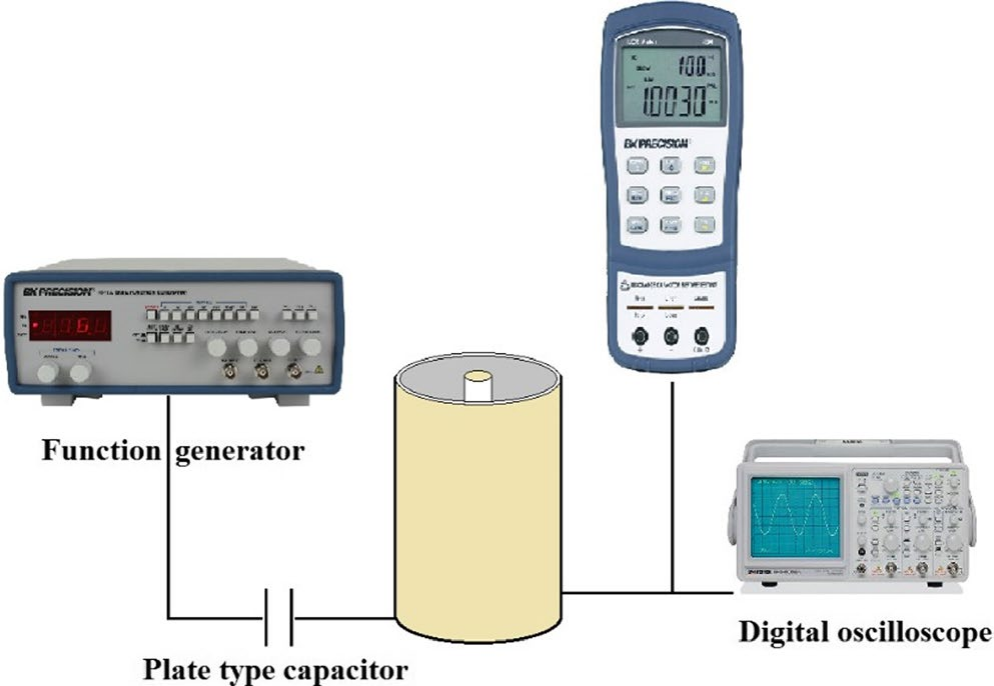
Block diagram of instrument for measuring the dielectric constant of wheat seeds and straw

The cylindrical capacitor with a specified capacity was placed in a series circuit connected to an alternating source with a sine wave at 20 V and a frequency range of 1, 10, 20, 100, and 500 kHz (Figure 3). The voltage of capacitor was measured in different situations with dielectric of air (empty) and two wheat varieties Mehregan and Chamran at 5 levels of moisture contents (from 8% to 30%), and the straw of the cluster of these two varieties.

**Figure 4:**
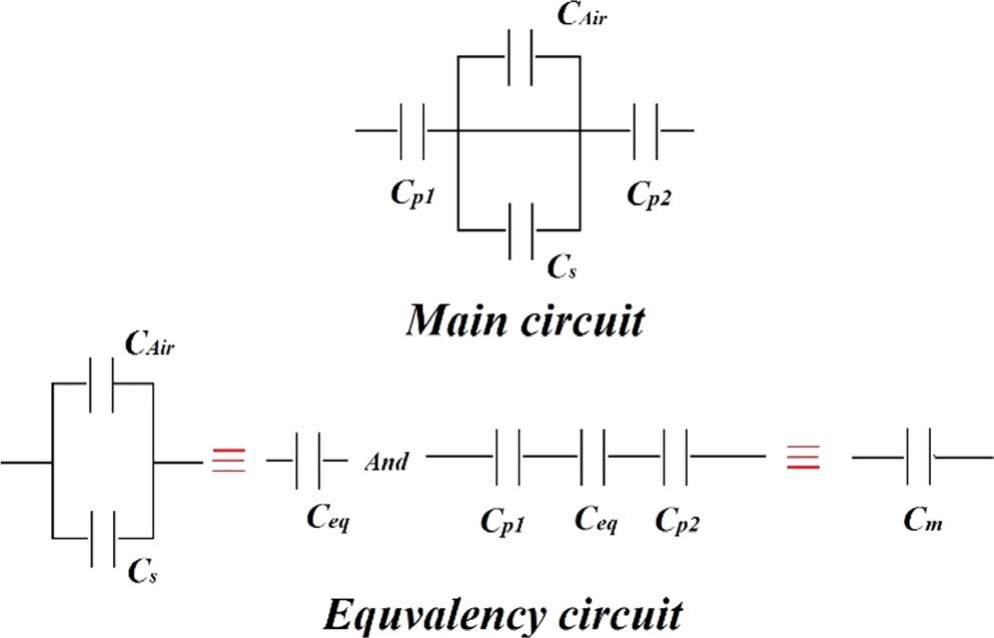
The equivalent circuit of capacitive sensor

The capacitance of a cylindrical capacitor can be calculated by Equation (1).

(1) $$C=2\pi \varepsilon_{r}\varepsilon_{0}h/Ln\left(\frac{b}{a}\right)$$

where *ε*_0_ is the vacuum permittivity*, ε_r_* is the dielectric constant of material, *h* is the height of material that is placed in the capacitive cylinder, *b* and *a* are the outer and inner radius of cylinder, respectively (Soltani & Alimardani, 2011b). It can be seen that on both sides of the cylinder, polyethylene coating is in contact with the electrode and the material with 1.5 mm thickness, so the capacitance of the polyethylene was measured in series with the system. The equivalent circuit diagram is shown in Figure 4.

In Figure 4, *C_m_* is the measured capacitance, *C_P_*_1_ and *C_P_*_2_ are the polyethylene capacitance, and *C_eq_* is the equivalent capacitance of the sample (*C_s_*) and air gap (*C_air_*) that existed among the grain and straw mass in the container. *C_eq_* is the sum of the capacity of the material and the air contained in its porosity space which is series with polyethylene coatings and can be calculated by Equations (2) and (3).

(2) $$C_{\mathrm{eq}}=\frac{1}{\frac{1}{C_{m}}-\frac{1}{C_{p_{1}}}-\frac{1}{C_{p_{2}}}}$$

(3) $$C_{\mathrm{eq}}=C_{s}+C_{\mathrm{air}}$$

If the ratio of air gap volume to the total volume of capacitor that was filled by the material (wheat and straw) is defined as the porosity (*P*), then the height of air gap (*h_a_*) is *P × h* and the height of materials in capacitor is (1 − *P*) × *h*. Therefore, the capacitance of air and materials and their dielectric coefficient can be calculated with the following equations.

(4) $$C_{\mathrm{air}}=\frac{2\pi \varepsilon_{0}Ph}{Ln\left(\frac{b}{a}\right)}$$

(5) $$C_{s}=C_{\mathrm{eq}}-C_{\mathrm{air}}$$

(6) $$\varepsilon_{s}=\frac{C_{s}Ln\frac{b}{a}}{2\pi \varepsilon_{0}\left(1-P\right)h}$$

#### 2.2.2 Moisture content

The moisture content of samples was calculated on dry basis using Equation (7). Average porosity of wheat and straw are 0.45 were 0.64, respectively.

(7) $$\%MC_{\mathrm{db}}=\frac{w_{w}}{w_{d}}\times 100=\frac{\left(w_{i}-w_{d}\right)}{w_{d}}$$

After measuring the moisture content of the samples (grain particles and straw) and transferring them to the cylindrical capacitor, the capacitor was placed in the circuit according to the Figures 2 and 3 so the electrical current and voltage of the capacitor were measured at 5 levels of moisture content for grain and 1 level for straw at 1, 10, 20, 100, and 500 kHz frequencies based on Guo, Yang, Zhu, Wang, and Guo (2013). The capacitance of circuit elements was calculated according to Equations (1), (2), (3), (4), (5). Therefore, the dielectric constant was measured at each moisture content and frequency according to Equation (6). Data analyses of the dielectric constant were carried out using a Factorial experiment in a Complete Randomized Design (CRD) with tree replications. Microsoft SAS (Ver. 9) was used for data analyses and Microsoft Excel 2016 was used to determine the regression models between dielectric constant and moisture content at different frequencies. The dielectric constant (*ε_r_*) and the moisture content (*Mc* %) data of different materials (*V*_1_, *V*_2_, and *V*_3_) in different frequencies were fitted to power, exponential, and polynomial models. The models were evaluated according to the statistical criterion *R* ^2^ for verifying the goodness of fit. The best model with the highest *R* ^2^ was selected to predict the *ε_r_* of materials as a function of the moisture content. Table 1 shows the symbols used for materials, moisture contents, and frequencies.

**Table 1:** Symbols used for materials, moisture contents and frequencies

Material		Moisture content		Frequency	
Chamran wheat grain crop	*V*_1_	8%	*M*_1_	1 kHz	*F*_1_
Mehregan wheat grain crop	*V*_2_	14%	*M*_2_	10 kHz	*F*_2_
Straw of two crop	*V*_3_	20%	*M*_3_	20 kHz	*F*_3_
		25%	*M*_4_	100 kHz	*F*_4_
				500 kHz	*F*_5_

## 3 RESULTS AND DISCUSSION

**Figure 5:**
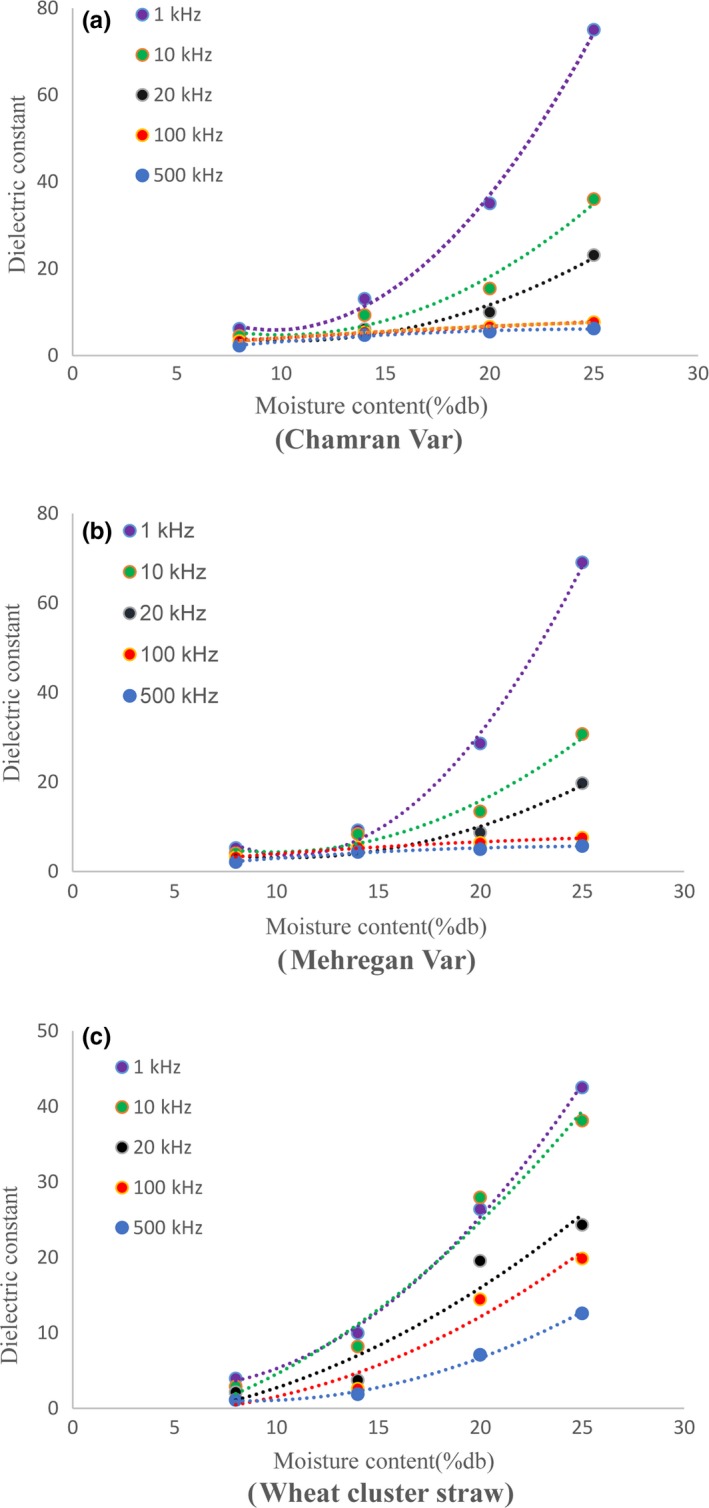
Change of dielectric constant versus 4 levels of moisture content for (a) Chamran variety, (b) Mehregan variety, and (c) wheat cluster straw

The obtained dielectric constants of wheat grain and cluster straw samples at 25°C and four moisture content levels and frequency range from 1 to 500 kHz are shown in Figure 5. Results showed that increasing moisture content caused an increase in dielectric constant in both wheat grain and cluster straw. This increase was more significant at the frequency of 1 kHz, and the increase in frequency decreased this constant, and it was higher at higher moisture contents, especially for grains.

Results of the polynomial model of dielectric constant as a function of moisture content (% *db*) fitted by regression analysis to the experimental data in 5 levels of frequency using the general quadratic equation as: *ε_r_* = *a*(*Mc*_(%_ *_db_*_)_)^2^ + *b*(*Mc*_(%_ *_db_*_)_) + *c* based on model parameters (*a*, *b*, and *c*) and *R* ^2^ are given in Table 2. Results show that at all frequencies, there is a high correlation between dielectric constant and moisture content. Factorial experiment in a randomized complete block design was used to analyses the result obtained from different moisture contents and frequencies. Table 3 shows the results of analyses of variance.

**Table 2:** Equations coefficients of the quadratic model of dielectric constant

Frequency	*V*_1_		*V*_2_		*V*_3_	
*F*_1_	*a*	8.27	*a*	9.15	*a*	2.53
	*b*	−18.50	*b*	−24.65	*b*	0.57
	*c*	16.53	*c*	21.02	*c*	0.32
	*R* ^2^	.99	*R* ^2^	.99	*R* ^2^	.99
*F*_2_	*a*	3.94	*a*	3.25	*a*	1.21
	*b*	−9.64	*b*	−7.73	*b*	6.51
	*c*	10.87	*c*	901,138	*c*	−6.02
	*R* ^2^	.98	*R* ^2^	.98	*R* ^2^	.96
*F*_3_	*a*	2.60	*a*	2.81	*a*	0.79
	*b*	−6.64	*b*	−5.70	*b*	4.29
	*c*	7.80	*c*	6.90	*c*	−4.20
	*R* ^2^	.99	*R* ^2^	.98	*R* ^2^	.91
*F*_4_	*a*	−0.25	*a*	−2.61	*a*	0.99
	*b*	2.65	*b*	2.71	*b*	1.83
	*c*	0.97	*c*	0.80	*c*	−2.49
	*R* ^2^	.99	*R* ^2^	.99	*R* ^2^	.94
*F*_5_	*a*	−0.42	*a*	−0.38	*a*	1.18
	*b*	3.36	*b*	−3.06	*b*	−1.92
	*c*	−0.57	*c*	−0.47	*c*	1.65
	*R* ^2^	.98	*R* ^2^	.98	*R* ^2^	.99

**Table 3:** Analyses of variance of wheat and straw cultivars dielectric constant (*ε_r_*)

Sources of variables	*df*	Sum of square	Mean square	*F* value	Pr > *F*
Materials	2	95.35	47.67	137.28	<0.0001
Moisture content	3	16,459.85	5,486.62	1,597.80	<0.0001
Frequency	4	11,410.04	2,852.51	8,213.31	<0.0001
Materials × Moisture	6	403.60	67.26	193.68	<0.0001
Materials × Frequency	8	1,085.18	135.64	390.57	<0.0001
Moisture × Frequency	12	9,474.62	789.55	2,273.38	<0.0001
Materials × Moisture × Frequency	24	1,660.35	69.18	199.20	<0.0001
CV	4.44				

Results of the analysis of variance show that the dielectric constant in both wheat grain cultivars had a significant difference in 1% level, and this difference was most significant between grains and cluster straw. The effect of moisture content was significant at 1% level on the dielectric constant, and the frequency up to 500 kHz had significant effects on the dielectric constant. Interactions of parameters (material × moisture content, material × frequency, and moisture content × frequency) were also significant at 1% level.

Tables 4 and 5 show the effect of wheat and straw cultivars, moisture content, frequency, and their interactions on dielectric constant. The most changes in dielectric constant in the variable moisture content levels were for cluster straw particles with 2.25 and 27.48 in 8% and 25%, respectively. Maximum and minimum of grain's dielectric constant for both cultivars were in 25 Mc% and 8 Mc% equal to 27 and 3.7, respectively. The effect of increasing the frequency on the reduction of the dielectric constant in wheat grain particles (both varieties) was higher than on straw particles. Maximum and minimum values of dielectric constant in frequency varying were 32.33 and 4.3 for *V*_1_ at 1 kHz and *V*_2_ at 500 kHz, respectively.

**Table 4:** Comparison of the effect of wheat and straw cultivars, moisture content, frequency, and their interactions on dielectric constant (*ε_r_*)

Dielectric constant					
Material	*V*_1_	*V*_2_	*V*_3_		
	13.98^a^	12.26^c^	13.53^b^		
Moisture content	*M*_1_	*M*_2_	*M*_3_	*M*_4_	
	3.24^d^	6.5^c^	15.34^b^	27.97^a^	
Frequency	*F*_1_	*F*_2_	*F*_3_	*F*_4_	*F*_5_
	27.02^a^	16.64^b^	10.76^c^	6.97^d^	4.88^e^

Note

Means with the same letter are not significantly different.

**Table 5:** Comparison the effects of interactions of wheat and straw cultivars, moisture content, and frequency on dielectric constant (*ε_r_*)

	Dielectric constant											
Material × Moisture	*V*_1_ *M*_1_	*V*_2_ *M*_1_	*V*_3_ *M*_1_	*V*_1_ *M*_2_	*V*_2_ *M*_2_	*V*_3_ *M*_2_	*V*_1_ *M*_3_	*V*_2_ *M*_3_	*V*_3_ *M*_3_	*V*_1_ *M*_4_	*V*_2_ *M*_4_	*V*_3_ *M*_4_
	3.93^j^	3.54^j^	2.25^k^	7.7^g^	6.52^h^	5.28^i^	14.51^e^	12.42^f^	19.1^d^	29.76^a^	26.57^c^	27.48^b^
Material × Frequency	*V*_1_ *F*_1_	*V*_2_ *F*_1_	*V*_3_ *F*_1_	*V*_1_ *F*_2_	*V*_2_ *F*_2_	*V*_3_ *F*_2_	*V*_1_ *F*_3_	*V*_2_ *F*_3_	*V*_3_ *F*_3_			
	32.33^a^	28.03^b^	20.71^c^	16.49^e^	14.15^f^	19.3^d^	10.65^h^	9.21^i^	12.44			
	*V*_1_ *F*_4_	*V*_2_ *F*_4_	*V*_3_ *F*_4_	*V*_1_ *F*_5_	*V*_2_ *F*_5_	*V*_3_ *F*_5_						
	5.75^j^	5.63^j^	9.53^i^	4.67^k^	4.3^k^	5.67^j^						
Moisture × Frequency	*M*_1_ *F*_1_	*M*_2_ *F*_1_	*M*_3_ *F*_1_	*M*_4_ *F*_1_	*M*_1_ *F*_2_	*M*_2_ *F*_2_	*M*_3_ *F*_2_	*M*_4_ *F*_2_	*M*_1_ *F*_3_	*M*_2_ *F*_3_		
	5.12^L^	10.74^h^	30.01^c^	62.24^a^	3.82^n^	8.63^ij^	18.94^e^	35.18^b^	2.85^o^	5.06^L^		
	*M*_3_ *F*_3_	*M*_4_ *F*_3_	*M*_1_ *F*_4_	*M*_2_ *F*_4_	*M*_3_ *F*_4_	*M*_4_ *F*_4_	*M*_1_ *F*_5_	*M*_2_ *F*_5_	*M*_3_ *F*_5_	*M*_4_ *F*_5_		
	12.75^f^	22.41^d^	2.57^o^	4.46^m^	9.15^i^	11.7^g^	1.85^p^	3.65^n^	5.87^k^	8.17^j^		

Note

Means with the same letter are not significantly different.

These results were similar to the results of other researches. Nelson, Guo, Trabelsi, and Kays (2007) reported a reduction in the dielectric constant for wheat grain at a frequency range of 10–1,000 MHz. Kardjilova, Rangelov, and Hlavacova (2013) obtained similar results for spelled grains—*T. dicoccum* in the frequency range of 20–200 kHz in a moisture content of 11.4%, reducing the dielectric constant, conductivity, and capacitance of the particle. Guo et al. (2013) reported the increase of dielectric constant of straw by increasing *Mc*% from 10% to 20%. Similar results were obtained by Berber et al. (2001) in reducing the dielectric constant for coffee beans at 11%–12% moisture content and a frequency range of 500–5,000 kHz.

## 4 CONCLUSION

Increasing moisture content in most agricultural products due to the presence of water molecules in the particles that have a high dielectric coefficient and high electrical conductivity reduces electrical capacities and increase electrical conductivity and their dielectric constant. This phenomenon is more significant in low frequencies. However, this process tends in straw to be faster than wheat grain particles, so making it difficult to separate straw and grain in electrical fields at straws with moisture content more than 12%.

## CONFLICT OF INTEREST

The authors declare that they do not have any conflict of interest.

## ETHICAL APPROVAL

This study does not involve any human or animal testing.
